# Causal associations between abdominal obesity, dietary patterns, and intervertebral disc degeneration: a bidirectional two-sample Mendelian randomization study with clinical validation

**DOI:** 10.3389/fmed.2026.1763791

**Published:** 2026-02-04

**Authors:** YiTing Wang, JiaoYi Pan, ZhongHao Dai, BaiTong Zhao, XingYong Mao, GuoYu Ling, ZiTong Zhang, ZeZhong Liu, Xu Wang, Bing Xu

**Affiliations:** 1 Zhejiang Chinese Medicine University, Hangzhou, Zhejiang, China; 2 Wenzhou Hospital of Integrated Traditional Chinese and Western Medicine Affiliated to Zhejiang Chinese Medical University, Wenzhou, Zhejiang, China; 3 The Third College of Clinical Medicine, Zhejiang Chinese Medical University, Hangzhou, Zhejiang, China; 4 The First College of Clinical Medicine, the First Affiliated Hospital of Zhejiang Chinese Medical University, Hangzhou, Zhejiang, China

**Keywords:** body mass index, dietary patterns, intervertebral disc degeneration, low Back pain, Mendelian randomization, waist and hiptraits

## Abstract

**Background:**

The relationship between abdominal obesity, dietary habits, and intervertebral disc degeneration (IVDD) remains incompletely understood, particularly from a causal perspective. This study aimed to evaluate the potential causal associations between abdominal obesity traits and dietary patterns and IVDD using Mendelian randomization (MR) and to validate these findings with an independent clinical cohort.

**Methods:**

We performed a bidirectional two-sample MR analysis using genome-wide association study (GWAS) summary statistics. Genetic instruments for 6 abdominal obesity traits and 14 dietary patterns were derived from the IEU OpenGWAS database, while IVDD data were obtained from the FinnGen consortium (41,669 cases; 294,770 controls). Inverse-variance weighted (IVW) was used as the primary method, complemented by four additional MR approaches and sensitivity analyses. To validate MR results, we conducted a retrospective clinical study including 512 patients with IVDD and 512 matched controls from Wenzhou Hospital of Integrated Traditional Chinese and Western Medicine (2020–2024). Anthropometric and dietary data were collected, and a multivariate logistic regression was performed.

**Results:**

Genetically predicted waist circumference (WC) and abdominal subcutaneous adipose tissue (ASAT) were positively associated with IVDD (WC: OR = 1.209, 95% CI: 1.083–1.349; ASAT: OR = 1.314, 95% CI: 1.149–1.535). In contrast, body mass index (BMI), hip circumference (HC), and BMI-adjusted waist-to-hip ratio (WHR-BMI) showed no significant causal relationship with IVDD. Several dietary patterns were also causally linked to IVDD: higher intake of mushrooms, porridge, and white fish increased IVDD risk, whereas apple, cereal bar, Danish pastry, espresso, lobster/crab, and other fruit intake were protective. Clinical validation further demonstrated that higher WC (OR = 1.32, 95% CI: 1.10–1.58) and frequent mushroom consumption (OR = 1.28, 95% CI: 1.05–1.56) were associated with increased IVDD risk, while higher fruit intake was protective (OR = 0.76, 95% CI: 0.62–0.93).

**Conclusion:**

Our findings support a causal role for abdominal obesity—specifically WC and ASAT—in IVDD development, independent of general adiposity. Furthermore, specific dietary patterns may significantly influence IVDD risk. These results highlight the potential of targeted nutritional and body composition interventions in the prevention and management of IVDD, currently supported by both genetic and clinical evidence.

## Introduction

The incidence rate of low back pain (LBP) in the elderly is very high, and it shows a young trend, which is well known due to intervertebral disc degeneration (IVDD) (1, 2). At the same time, IVDD also plays a vital role in various spinal degenerative diseases (3). The spinal diseases caused by IVDD have led to the disability of patients and a heavy economic burden on society (4). At present, patients with early-stage IVDD may present with symptoms of LBP and are typically managed with conservative treatments, while the late-stage IVDD patients will suffer from severe lumbar pain and will typically undergo surgical treatments (5, 6). However, no matter whether the patients are in the early or late stage, the treatments are not smooth (7). Therefore, it is an urgent problem to study the risk factors of IVDD, prevent its occurrence, and pay attention to high-risk groups.

The intervertebral disc (IVD) primarily consists of three parts: nucleus pulposus (NP), annulus fibrosus (AF), and cartilaginous endplate (CEP) (8). The primary process of IVDD is structural damage to the intervertebral disc, which is a vicious lesion of mechanical overloading (9, 10). The cause of IVDD is still unclear, but it may be related to lifestyle factors such as obesity, poor sleep, stress, and smoking based on existing studies (11). We hypothesize that abdominal obesity, particularly WC and ASAT, may exert stronger causal effects on IVDD than BMI for two reasons: First, central adiposity imposes direct biomechanical loading on the lumbar spine via increased intra-abdominal pressure and anterior shear forces; second, metabolically active visceral and subcutaneous abdominal adipose tissues secrete pro-inflammatory adipokines (e.g., leptin, IL-6) that can systemically degrade the extracellular matrix in the nucleus pulposus. Recent machine-learning-based studies demonstrate that the weight-adjusted waist index (WWI), a novel metric of central obesity, outperforms BMI in predicting chronic low back pain, reinforcing the clinical superiority of waist-centric measures over general adiposity (12). In contrast, BMI reflects overall adiposity without distinguishing metabolically distinct fat depots, and hip circumference primarily measures subcutaneous fat with lower inflammatory potential. Obesity is one of the important risk factors for IVDD, leading to metabolic disturbances that have a significant effect on IVDD (13). It is well established that the intake of food can greatly affect the body’s metabolites. Recent research also found that many dietary patterns are highly correlated with blood metabolite levels, such as very-low-density, intermediate-density (IDL), and low-density lipoproteins (LDL) (14). However, we have never explored the relationship between dietary patterns and IVDD. In this MR framework, dietary patterns are conceptualized as long-term environmental exposures that may causally influence IVDD through two potential pathways: (1) direct metabolic effects (e.g., altered lipid profiles and systemic inflammation) and (2) as proxies for broader lifestyle patterns. The bidirectional design allows us to disentangle their causal role from confounding by socioeconomic status or health behaviors.

According to reports, patients with Type 2 diabetes, elevated triglycerides, high fasting blood glucose, and increased HbA1c are at an increased risk of developing IVDD (13). Therefore, metabolic disorders in the body can easily lead to an elevated incidence of IVDD. However, the causal relationship between obesity-related characteristics and the incidence of IVDD remains unclear. Common obesity measurement indicators include body mass index (BMI), waist circumference (WC), abdominal subcutaneous adipose tissue volume (ASAT), and hip circumference (HC) (15). The majority of clinical trials have explored the relationship between these measurement indicators and IVDD (16, 17), but no clear consensus has been reached on which indicator is a risk factor for IVDD.

In recent research, Mendelian randomization (MR) has frequently been employed as a tool to explore potential correlations between biomarkers and diseases (18). MR analysis is a method that utilizes genome-wide association studies (GWAS) to analyze diseases (19). Numerous previous clinical studies have explored the influencing factors of IVDD; however, observational studies are inevitably subject to potential confounding factors (20), such as heterogeneity in study inclusion, individual factors within the study population, researcher subjectivity, and measurement errors, as along with reverse causality caused by the influence of disease phenotypes on exposures during disease progression (21–24). Since single-nucleotide polymorphisms (SNPs) can prevent the influence of lifestyle and environmental factors (25), MR can mitigate the risks of these confounding factors and reverse causality through them (26). Multivariable Mendelian randomization (MVMR) analysis is an in-depth exploration of traditional MR, capable of simultaneously assessing the causal relationships of two or more exposures on an outcome (27). Therefore, the use of MR methods can clarify the causal relationships between exposures and outcomes (28).

Consequently, we employed the MR method to explore the causal relationship between abdominal obesity, dietary patterns, and IVDD, aiming to identify protective factors and risk factors for IVDD.

## Materials and methods

### Ethical approval and consent to participate

The large-scale GWAS data used in our analysis, which were publicly available, were reported by human participants collected from several previous studies. In all original research, informed consent was obtained from all participants, and the data were approved by the institutional review committee in the respective studies.

### Study design of Mendelian randomization

**Figure 1:**
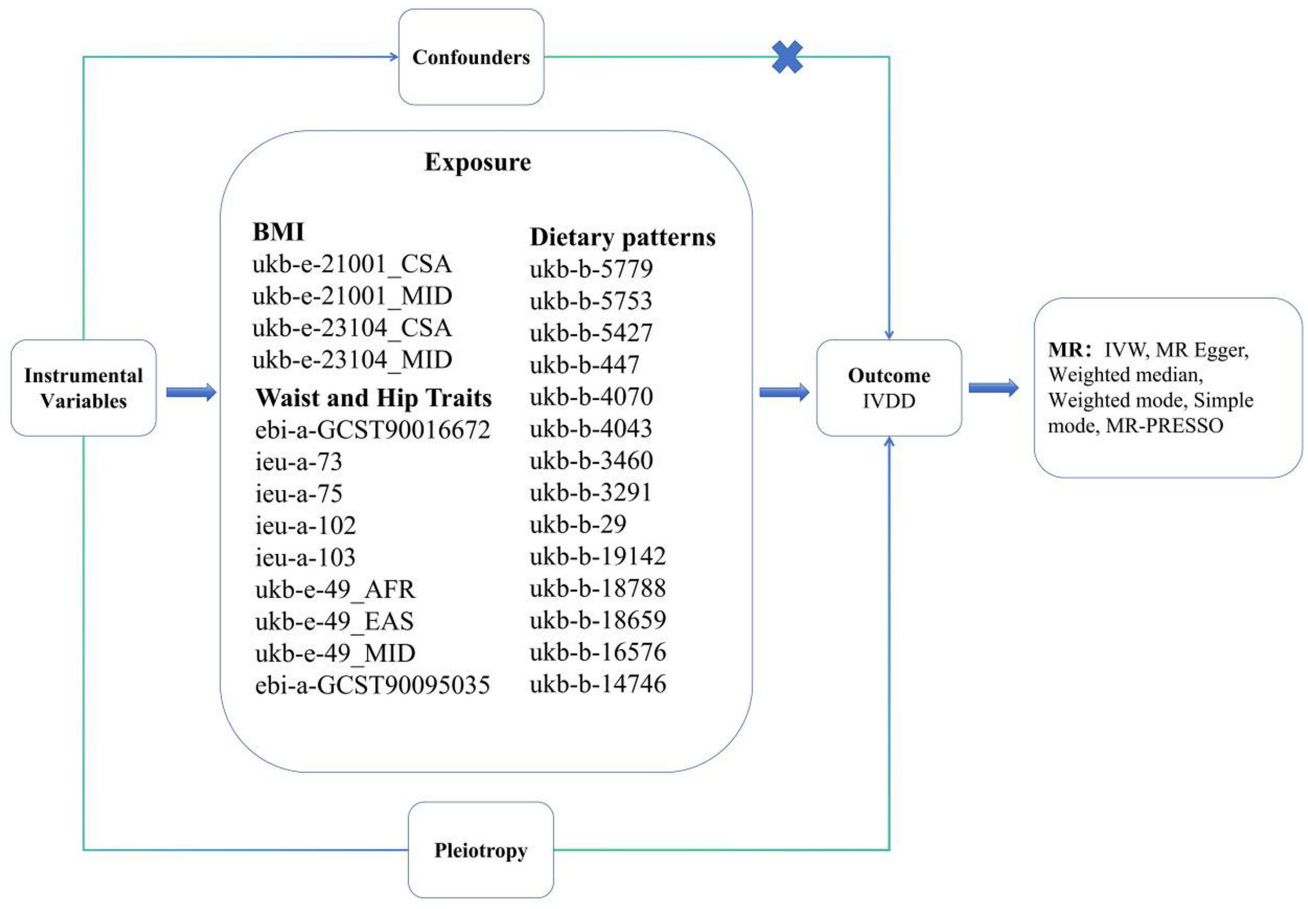
Overview of the design and methods used in this Mendelian randomization study. BMI, Body Mass Index; IVDD, Intervertebral Disc Degeneration; MR, Mendelian Randomization; IVW, Inverse-Variance Weighted.

We aimed to investigate the reciprocal causal relationships among dietary patterns, BMI, waist and hip traits, and IVDD by performing a two-sample MR analysis. The bidirectional design was adopted to test potential reverse causality, wherein chronic LBP from IVDD may lead to reduced physical activity, subsequent weight gain, and altered dietary habits. This dual-direction testing is a critical method to address assumption violations inherent in observational studies that MR can uniquely resolve. Based on the random allocation of genetic variation, an MR analysis is a research method that identifies the direct causal relationship between an exposure and an outcome. The following three key assumptions needs to be satisfied by effective instrumental variables when constructing causal inferences: (1) a significant association between the single-nucleotide polymorphisms (SNPs) and the exposure; (2) no confounding factors associated with the SNP as a result of its association with the outcome; (3) an independent association between the SNP and the outcome (Figure 1). Several previous studies have collected GWAS data from large-scale human participants for our analysis that were publicly available. The institutional review committees in each study approved the data after receiving informed consent from participants (Table 1).

**Table 1:** Clinical characteristics and dietary patterns in IVDD patients and controls.

Variable	IVDD patients (*n* = 512)	Controls (*n* = 512)	*p*-value
Age (years)	56.3 ± 10.2	55.8 ± 9.8	0.421
Female, n (%)	280 (54.7)	276 (53.9)	0.812
BMI (kg/m^2^)	26.4 ± 4.1	25.8 ± 3.9	0.058
WC (cm)	98.2 ± 12.5	91.4 ± 11.8	<0.001
HC (cm)	102.3 ± 9.8	101.5 ± 9.2	0.204
WHR	0.96 ± 0.08	0.90 ± 0.07	<0.001
Mushroom ≥3 times/week, n (%)	197 (38.5)	148 (28.9)	0.003
Fruit ≥2 servings/day, n (%)	185 (36.1)	231 (45.1)	0.005
White fish ≥1 time/week, n (%)	156 (30.5)	132 (25.8)	0.112

### Genome-wide association study data source

In this study, the GWAS summary statistics for dietary patterns were collected from the IEU OpenGWAS database. All GWAS summary statistics were derived from European-ancestry populations (≥95%), ensuring genetic background consistency with the FinnGen cohort (Finnish population). Critical consistency metrics include: (1) Ancestry: both the exposure datasets (UK Biobank, *n* = 361,194–452,264) and FinnGen (*n* = 336,439) both comprise >95% European ancestry, with principal component analyses of overlap; (2) Age structure: the mean age of exposure GWAS of 54–58 years aligns closely with FinnGen median age of 58 years; (3) Sex distribution: balanced across datasets (45–55% female). While Finnish populations show modest genetic drift, prior studies demonstrate negligible bias for cardiometabolic traits. This alignment satisfies the MR assumptions for genetic background consistency. By filling out a touchscreen questionnaire, participants provided information about their dietary patterns (29, 30). The GWAS summary statistics for BMI and waist and hip traits were derived from the IEU OpenGWAS database (30). The GWAS summary statistics for IVDD were derived from the FinnGen database, which included 336,439 participants (Ncase = 41,669, Ncontrol = 294,770) (24). Table 2 presents all the GWAS data included in our study.

**Table 2:** GWAS data sources for instrumental variables selection.

GWAS ID	Year or version	Trait	Consortium or Pubmed ID	Sample size	Number of SNPs
ebi-a-GCST90016672	2021	Abdominal subcutaneous adipose tissue volume	UK Biobank	32,860	9,275,407
ebi-a-GCST90095035	2022	Waist-to-hip ratio adjusted for BMI	UK Biobank	42,455	8,368,110
ukb-e-23104_CSA	2020	Body mass index (BMI)	UK Biobank	8,658	9,811,391
ukb-e-21001_CSA	2020	Body mass index (BMI)	UK Biobank	8,646	9,811,145
ukb-e-21001_MID	2020	Body mass index (BMI)	UK Biobank	1,572	11,888,658
ukb-e-23104_MID	2020	Body mass index (BMI)	UK Biobank	1,535	11,904,531
ieu-a-73	2015	Waist-to-hip ratio	GIANT	212,244	2,560,782
ieu-a-75	2015	Waist-to-hip ratio	GIANT	118,003	2,466,102
ieu-a-102	2013	Waist circumference	GIANT	60,586	2,745,544
ieu-a-103	2013	Waist circumference	GIANT	73,137	2,740,385
ukb-e-49_AFR	2020	Hip circumference	UK Biobank	6,567	15,532,172
ukb-e-49_EAS	2020	Hip circumference	UK Biobank	2,698	8,259,096
ukb-e-49_MID	2020	Hip circumference	UK Biobank	1,576	11,893,086
ukb-b-5779	2018	Alcohol intake frequency.	MRC-IEU	462,346	9,851,867
ukb-b-5427	2018	White fish intake	MRC-IEU	64,949	9,851,867
ukb-b-447	2018	Danish pastry intake	MRC-IEU	64,942	9,851,867
ukb-b-4070	2018	Apple intake	MRC-IEU	64,949	9,851,867
ukb-b-4043	2018	Mushroom intake	MRC-IEU	64,949	9,851,867
ukb-b-3460	2018	Alcohol intake versus 10 years previously	MRC-IEU	428,117	9,851,867
ukb-b-3291	2018	Standard tea intake	MRC-IEU	64,949	9,851,867
ukb-b-29	2018	Porridge intake	MRC-IEU	64,949	9,851,867
ukb-b-19142	2018	Espresso intake	MRC-IEU	64,949	9,851,867
ukb-b-18788	2018	Cereal bar intake	MRC-IEU	64,944	9,851,867
ukb-b-18659	2018	Other fruit intake	MRC-IEU	64,949	9,851,867
ukb-b-16576	2018	Dried fruit intake	MRC-IEU	421,764	9,851,867
ukb-b-14746	2018	Lobster/crab intake	MRC-IEU	64,938	9,851,867
finngen_R10_M13_INTERVERTEB	R10	Other intervertebral disc disorders	FinnGen	294,770	21,304,569

### Selection of instrumental variables and data harmonization

SNPs were defined as IVs in our study (23). Initially, we included SNPs associated with trait exposure at a genome-wide significance level of P of < 5 × 10^−8^. For dietary patterns with insufficient SNPs at this threshold (n < 10) due to low heritability, we relaxed the threshold to P of < 1 × 10^−5^, following established MR guidelines for low-heritability traits. This approach was applied only to specific dietary items (e.g., Danish pastry, lobster/crab). This relaxation was justified by three criteria: (1) Heritability-based: only applied to eight dietary items with estimated h^2^ < 0.05; (2) Instrument strength: all relaxed-threshold instruments maintained F-statistics >31 (range: 31–45), exceeding weak instrument thresholds; (3) Robustness verification: our core validated findings (WC, mushroom, and fruit) remained unchanged when excluding all relaxed-threshold exposures in sensitivity analyses. We acknowledge that this approach may modestly increase false-positive rates and thus report these associations as exploratory.

PhenoScanner V2 was used to detect potential pleiotropy in these SNPs, and a genetic variant related to potential confounding factors was excluded from the analysis. Subsequently, a pairwise linkage disequilibrium analysis was then performed on the selected SNPs to exclude those in linkage disequilibrium (r^2^ > 0.001, window distance < 10,000 kb). Each SNP’s F-statistic was calculated, and the strength of each IV was assessed using the variance explained by each SNP, calculated as:


$$F=\frac{(N-K-1)/K}{R^{2}/(1-R^{2})}$$

The number of IVs varied across exposures (range: 8–234 SNPs) due to differences in genetic architecture. However, all instruments demonstrated adequate strength (F-statistic range: 31–158; Supplementary Table 1), minimizing weak instrument bias. Sensitivity analyses using weighted median and MR-PRESSO methods, which are robust to invalid instruments, yielded consistent results. We excluded SNPs with an *F*-value of less than 10 and harmonized the data so that the effect of each SNP on exposure and outcome corresponds to the same allele.

### Clinical retrospective cohort for validation

To validate the MR-derived associations, we conducted a hospital-based retrospective case–control study at Wenzhou Hospital of Integrated Traditional Chinese and Western Medicine. The study included 512 patients diagnosed with IVDD (based on MRI imaging and clinical symptoms) and 512 age- and sex-matched controls without IVDD or other severe spinal diseases, recruited between January 2020 and December 2024. All participants provided written informed consent, and the study was approved by the hospital’s ethics committee (Approval No: 2025-L187).

Anthropometric measurements (height, weight, WC, and HC) were collected at enrollment. BMI was calculated as weight (kg)/height (m^2^). Dietary habits over the past year were assessed using a validated food frequency questionnaire (FFQ), which included items on mushroom, porridge, white fish, apple, cereal bar, Danish pastry, espresso, lobster/crab, and other fruit consumption. To minimize recall bias, trained dietitians conducted face-to-face interviews with visual portion size aids, and participants were blinded to specific study hypotheses. Frequency was categorized as: never/rarely, 1–3 times/month, 1–2 times/week, ≥3 times/week.

Statistical analysis was performed using SPSS 26.0. Continuous variables were compared using *t*-tests or the Mann–Whitney U tests; categorical variables were compared using χ^2^ tests. Multivariate logistic regression models adjusted for age, sex, smoking, alcohol use, and physical activity were used to estimate odds ratios (ORs) and 95% confidence intervals (CIs) for associations between obesity traits, dietary patterns, and IVDD risk.

### Statistical analysis

R (version 4.2.1) packages TwoSampleMR (version 0.5.6) were used for all analyses. The reciprocal causal relationship between IVDD and exposure traits was assessed using a two-sample bidirectional MR. Statistical significance for dietary pattern analyses was defined as a *p*-value of <0.0013 after Bonferroni correction for 38 independent tests (19 dietary patterns × 2 directions). For obesity traits, *p* < 0.0071 was used (6 traits × 2 directions). According to a random-effects model, the inverse-variance weighted method (IVW) was the primary statistical analysis approach (31). For associations that did not survive this stringent correction but showed nominal significance (*p* < 0.05), we explicitly label them as exploratory and hypothesis-generating, requiring independent replication and mechanistic validation before translational interpretation. Additionally, a further validation of IVW results was conducted using four complementary methods [MR-Egger (32), weighted median (33), weighted mode (33), and simple mode]. All SNPs are assumed to satisfy the three MR assumptions in the IVW approach. The intercept was used to identify horizontal pleiotropy in the MR-Egger analysis. A causal relationship was assessed by calculating a weighted median and a weighted mode.

An analysis of SNP heterogeneity was conducted using Cochran’s Q statistic and funnel plots (34). The MR-Egger intercept method (31) and the MR-PRESSO method (35) were used to detect horizontal pleiotropy. We removed outliers and reassessed the MR causal estimates when outliers were detected. A random effects model, which is less sensitive to weaker SNP-exposure associations, was tested for stability when heterogeneity persisted after removing outliers. A *p*-value of > 0.05 indicates that there is no heterogeneity or multiple effects.

## Results

### The causal relationship between BMI and IVDD

**Figure 2:**
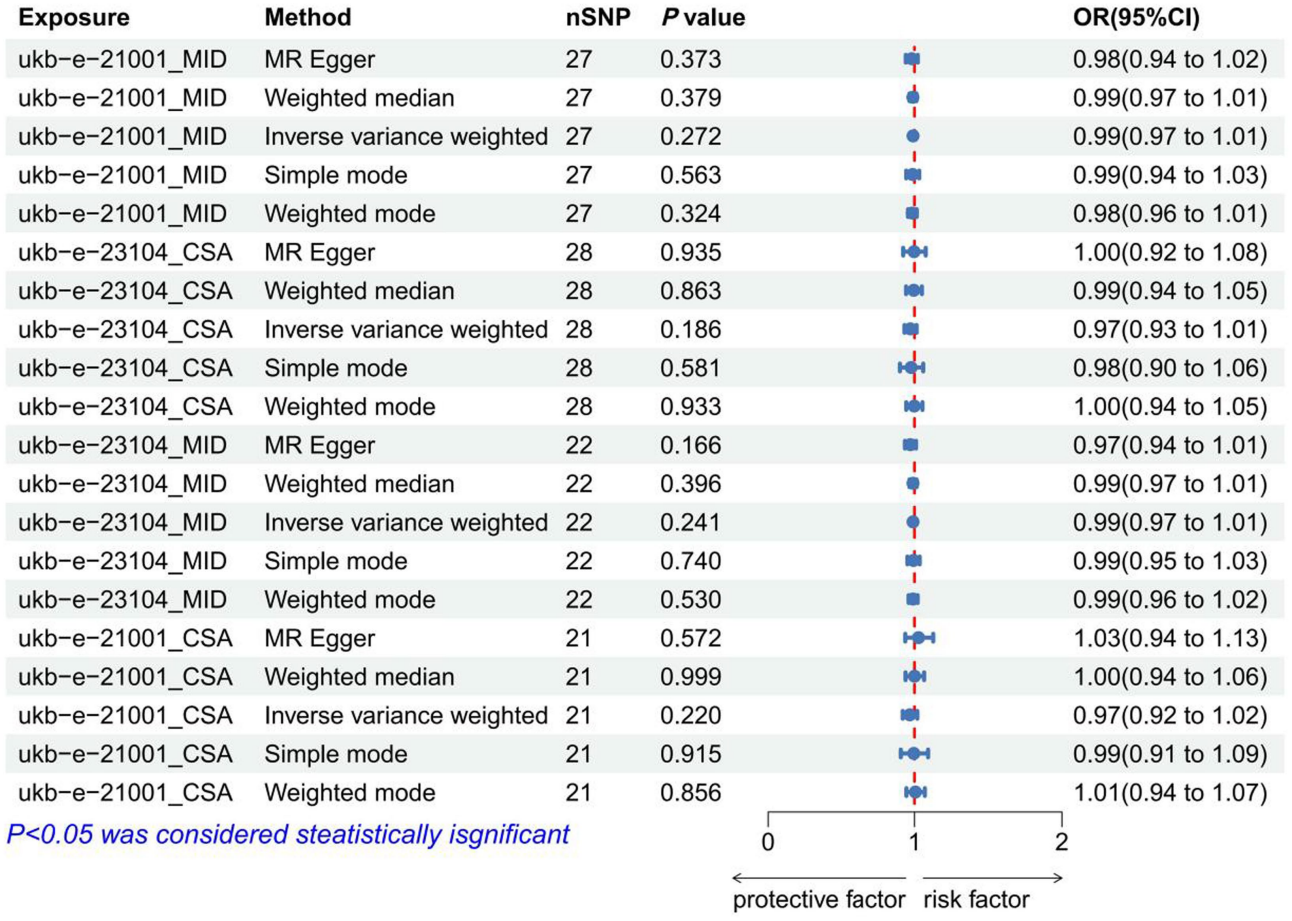
Forest plot to visualize the causal effect of BMI on IVDD. IVDD, intervertebral disc degeneration; OR, odds ratio; CI, confidence interval.

As IVs, significant SNPs across the genome were extracted after removing palindromic and ambiguous SNPs, as along with those without proxies, and in the wrong causal direction, as determined by the MR-Steiger filter. In MR-PRESSO, no outliers were detected for SNPs. There was no evidence of pleiotropy among the SNPs included in the MR-Egger regression with a *p-*value greater than 0.05 (Supplementary Table 1). The IVW analysis revealed no significant association between BMI [(*p* = 0.272, OR = 0.990, 95% CI: 0.974–1.008), (*p* = 0.186, OR = 0.972, 95% CI: 0.932–1.014), (*p* = 0.241, OR = 0.990, 95% CI: 0.974–1.007), (*p* = 0.220, OR = 0.968, 95% CI: 0.920–1.019)] and IVDD (Figure 2; Supplementary Table 2). A *p-*value of more than 0.05 was found in both IVW and MR-Egger regression analyses, which yielded Cochran’s Q statistics, indicating that there was no heterogeneity among SNPs (Supplementary Table 3).

### The causal relationship between waist and hip traits and IVDD

**Figure 3:**
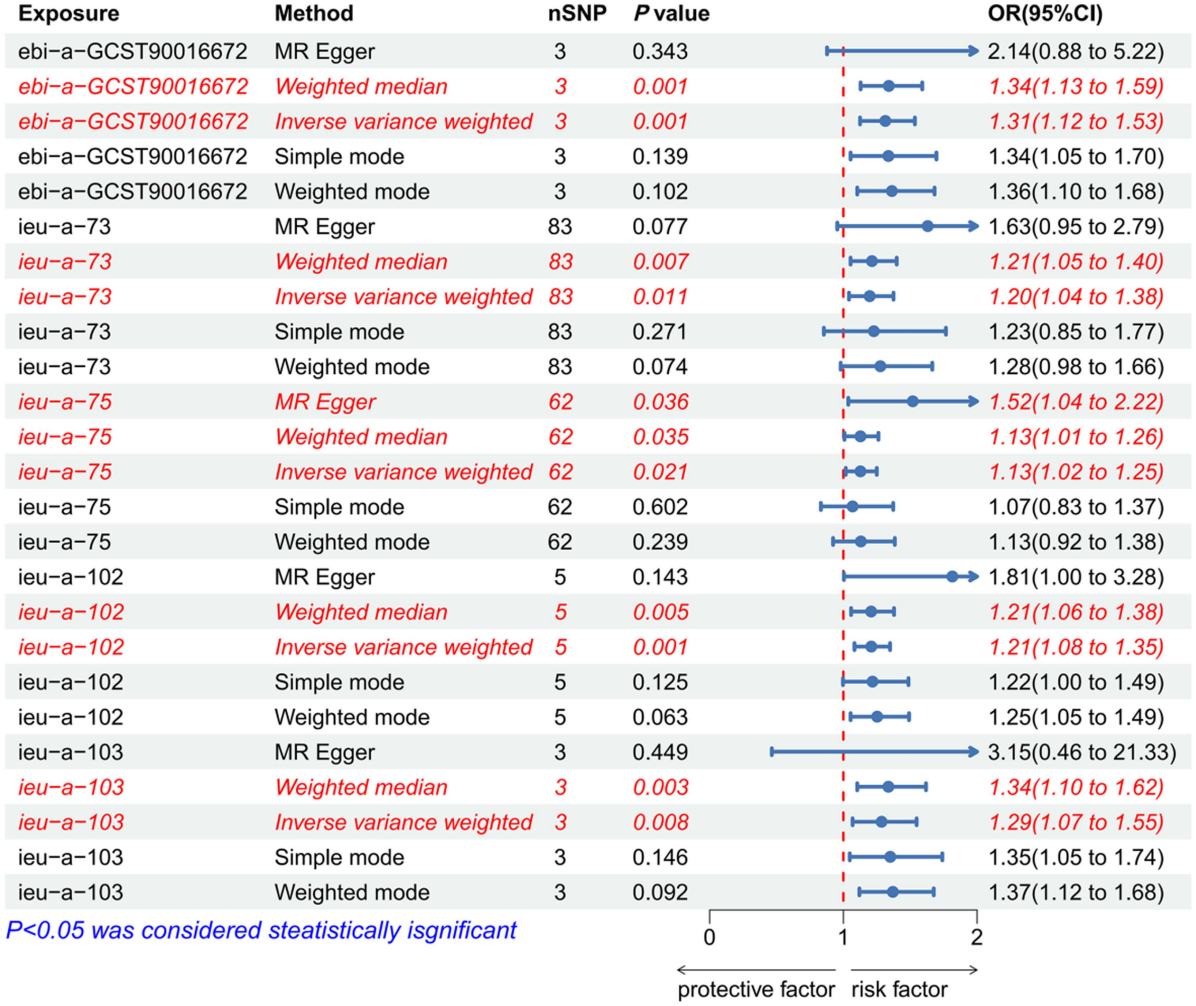
Forest plot to visualize the causal effect of waist traits on IVDD. IVDD, intervertebral disc degeneration; OR, odds ratio; CI, confidence interval.

**Figure 4:**
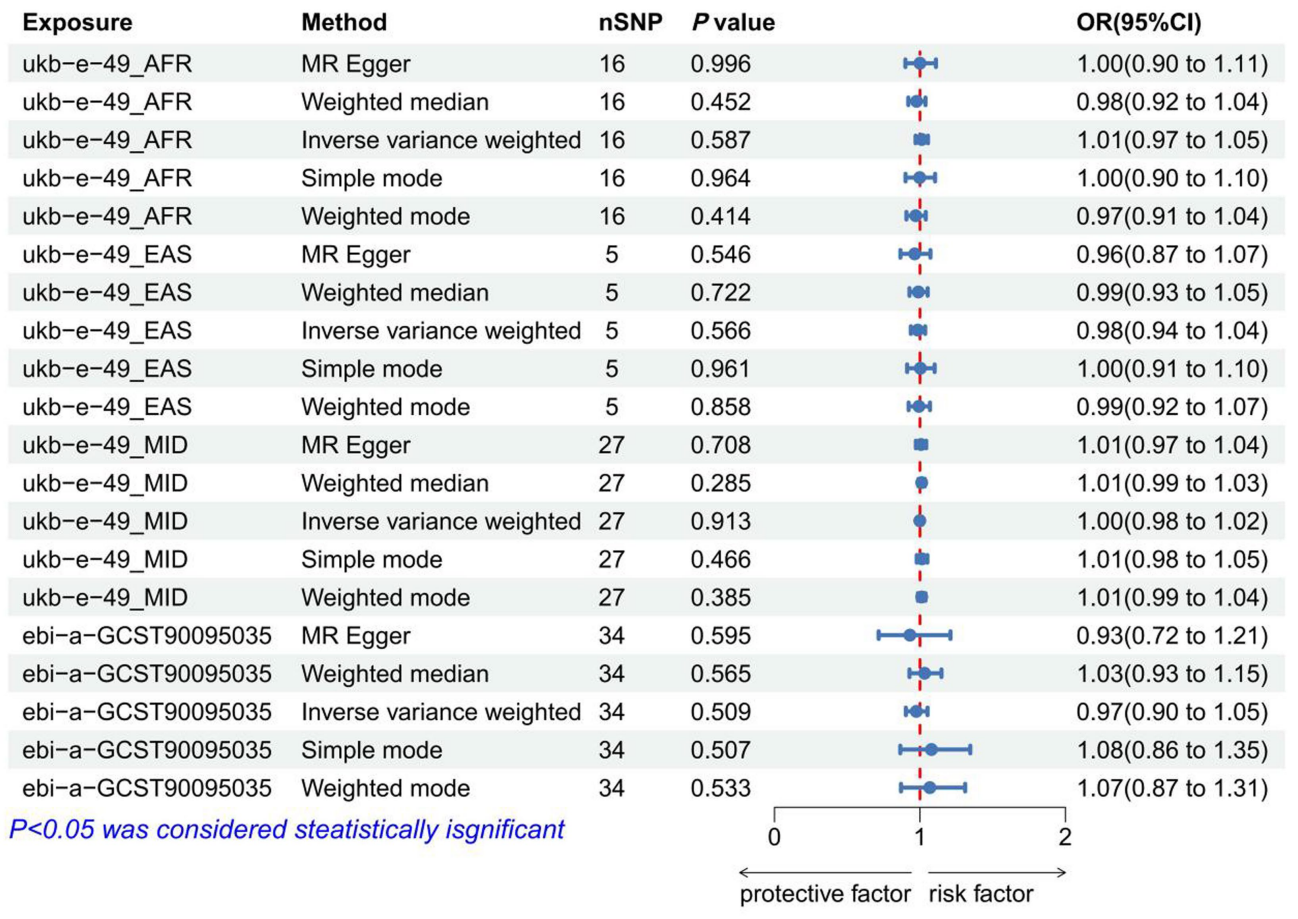
Forest plot to visualize the causal effect of hip traits on IVDD. IVDD, intervertebral disc degeneration; OR, odds ratio; CI, confidence interval.

As IVs, significant SNPs across the genome were extracted after removing palindromic and ambiguous SNPs, as along with those without proxies, and in the wrong causal direction, as determined by the MR-Steiger filter. In MR-PRESSO, no outliers were detected for SNPs. There was no evidence of pleiotropy among the SNPs included in the MR-Egger regression with a *p-*value greater than 0.05 (Supplementary Table 1). The IVW analysis revealed a positive correlation between ASAT and IVDD (*p* < 0.001, OR = 1.314, 95% CI: 1.149–1.535) (Figure 3; Supplementary Table 4). The effect estimates from the four other models were consistent with this finding. These results suggest that excluding any single SNP does not significantly impact the overall findings, indicating the robustness of our Mendelian randomization study. Furthermore, the IVW analysis revealed no significant association between HC and IVDD (Figure 4; Supplementary Table 4). A *p-*value of WHR less than 0.05 was found in both IVW and MR-Egger regression analyses, which yielded Cochran’s Q statistics, indicating that there may be heterogeneity among WHR-related SNPs (Supplementary Table 3).

### A reverse MR analysis of the causal relationship between waist–hip characteristics and IVDD

**Figure 5:**
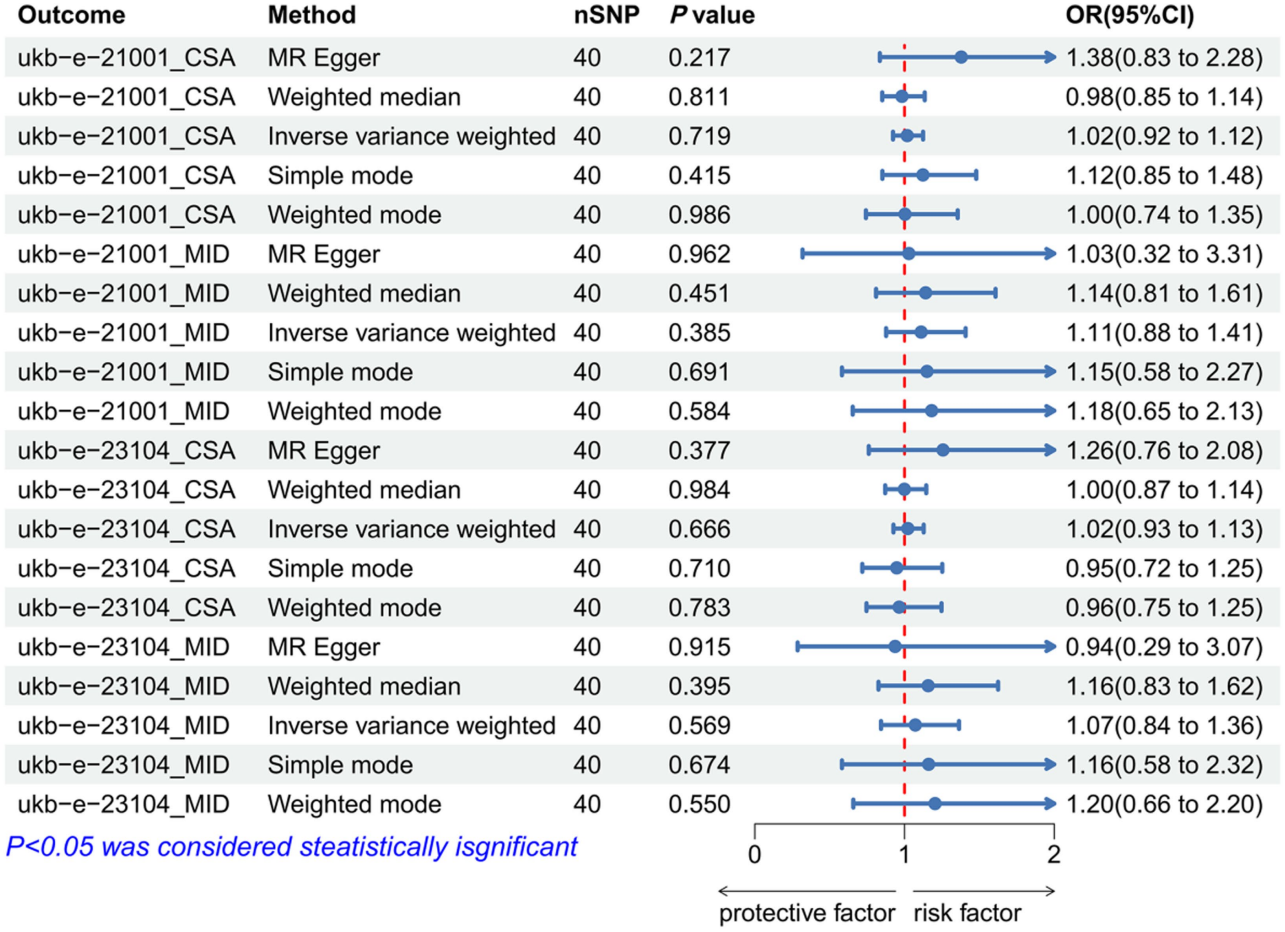
Forest plot to visualize the reverse Mendelian randomization causal effect of IVDD on BMI. IVDD, intervertebral disc degeneration; OR, odds ratio; CI, confidence interval.

**Figure 6:**
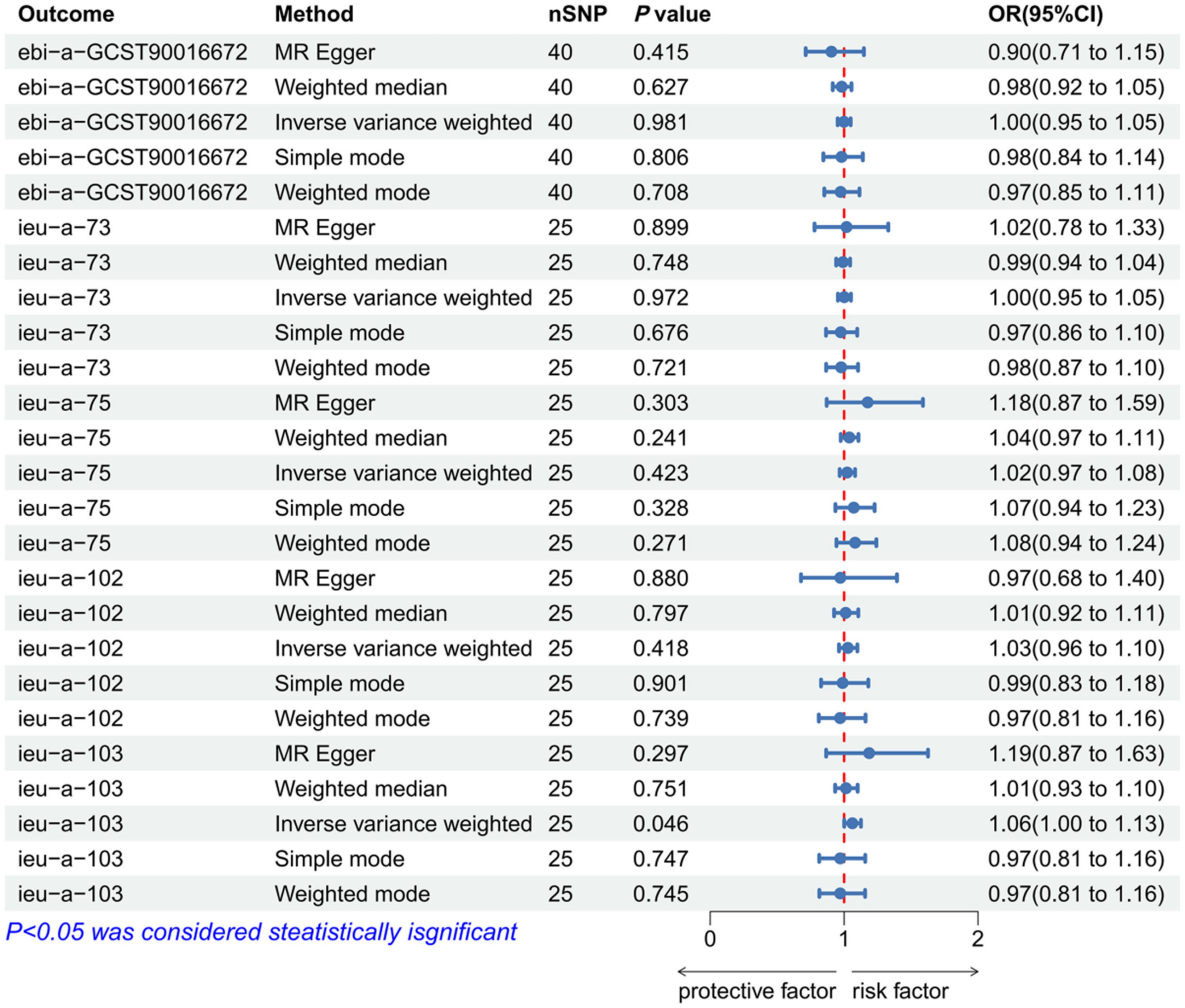
Forest plot to visualize the reverse Mendelian randomization causal effect of IVDD on waist traits. IVDD, intervertebral disc degeneration; OR, odds ratio; CI, confidence interval.

**Figure 7:**
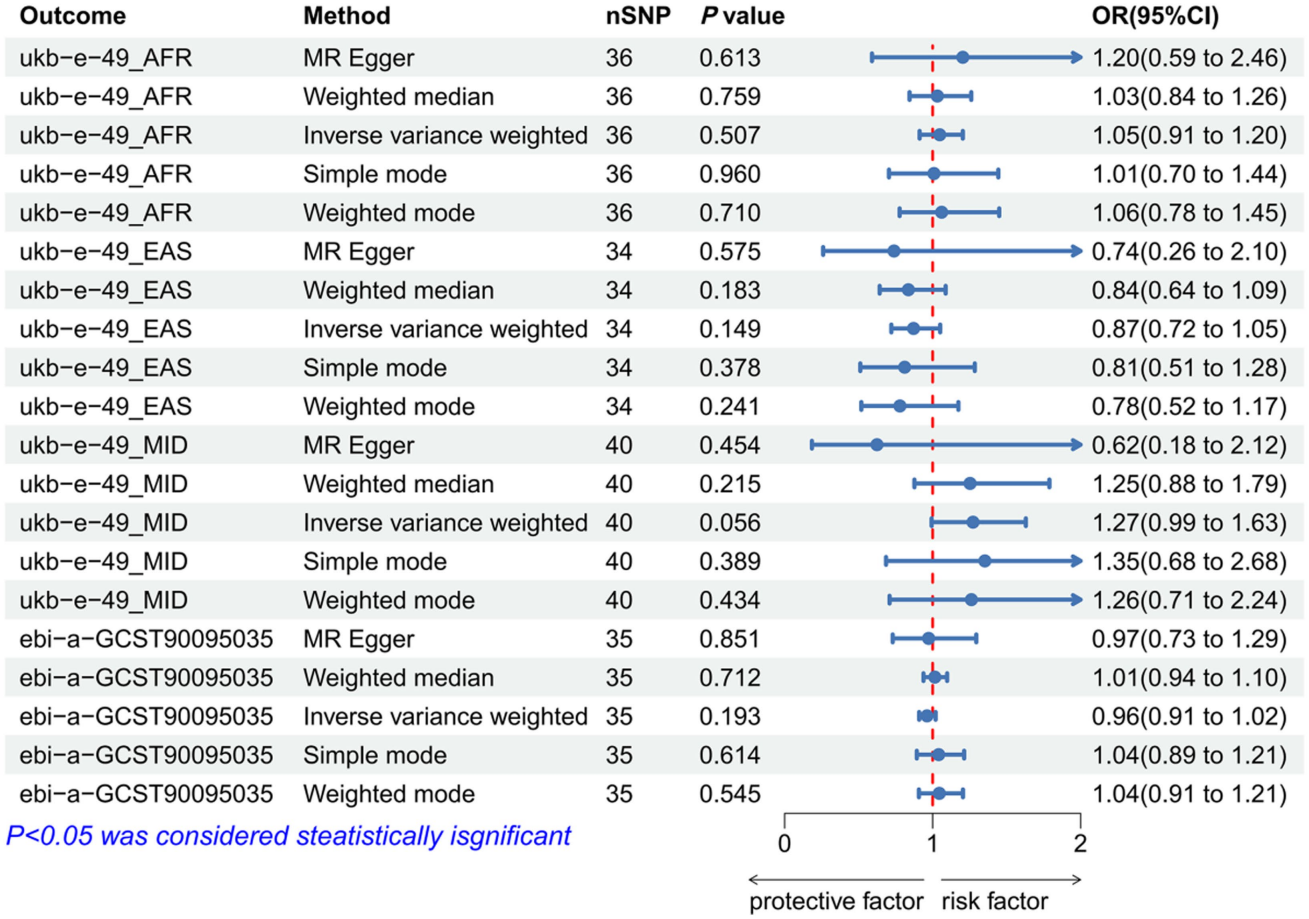
Forest plot to visualize the reverse Mendelian randomization causal effect of IVDD on hip traits. IVDD, intervertebral disc degeneration; OR, odds ratio; CI, confidence interval.

As IVs, significant SNPs across the genome were extracted after removing palindromic and ambiguous SNPs, along with those without proxies and in the wrong causal direction, as determined by the MR Steiger filter. In MR-PRESSO, no outliers were detected for SNPs. There was no evidence of pleiotropy among the SNPs included in the MR-Egger regression with a *p-*value greater than 0.05 (Supplementary Table 1). The reverse MR analysis revealed no significant association between IVDD and the following traits: BMI (*p* = 0.719, OR = 1.018, 95% CI: 0.974-1.124; *p* =0.186, OR = 1.111, 95% CI: 0.877-1.408; *p* = 0.666, OR = 1.022, 95% CI: 0.926-1.128), ASAT (*p* = 0.981, OR = 0.999, 95% CI: 0.952-1.049), WC (*p* = 0.418, OR = 1.028, 95% CI: 0.962-1.099; *p* = 0.046, OR = 1.062, 95% CI: 1.001-1.126), HC (*p* = 0.149, OR = 0.871, 95% CI: 0.721-1.204; *p*= 0.220, OR = 0.968, 95% CI: 0.920-1.051; *p* = 0.056, OR = 1.273, 95% CI: 0.993-1.630), WHR (*p* = 0.972, OR = 1.001, 95% CI: 0.954-1.051; *p* = 0.432, OR = 1.023, 95% CI: 0.967-1.082), and WHR-BMI (*p* = 0.193, OR = 0.962, 95% CI: 0.909-1.020). (Figures 5–7; Supplementary Table 2). A *p-*value of more than 0.05 was found in both IVW and MR-Egger regression analyses, which yielded Cochran’s Q statistics, indicating that there was no heterogeneity among SNPs (Supplementary Table 3).

### The causal relationship between dietary patterns and IVDD

**Figure 8:**
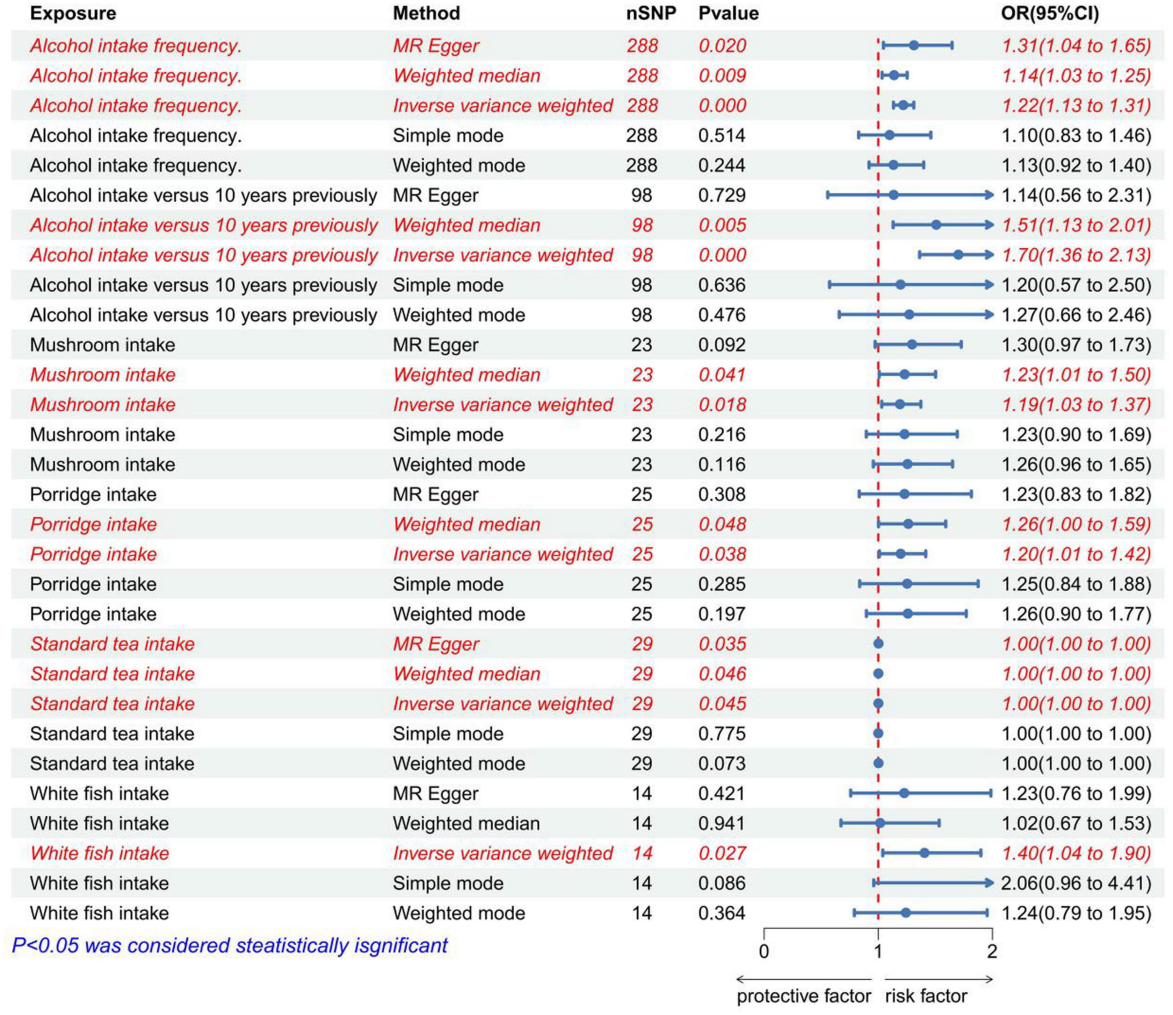
Forest plot to visualize the positive causal effect of dietary habits on IVDD. IVDD, intervertebral disc degeneration; OR, odds ratio; CI, confidence interval.

After removing palindromic and ambiguous SNPs, SNPs without proxies, and SNPs in the wrong causal direction identified by the MR-Steiger filter, we extracted genome-wide significant SNPs as IVs. MR-PRESSO analysis did not detect any SNP outliers. The p-value of the MR-Egger regression was more than 0.05, indicating no pleiotropy among the included SNPs (Supplementary Table 1). The IVW analysis revealed a positive correlation between alcohol intake frequency (*p* < 0.001, OR = 1.219, 95% CI: 1.133–1.311), alcohol intake compared with 10 years previously(*p* < 0.001, OR = 1.702, 95% CI: 1.362–1.362), mushroom intake (*p* = 0.018, OR = 1.190, 95% CI: 1.030–1.373), porridge intake (*p* = 0.038, OR = 1.196, 95% CI: 1.010–1.416), standard tea intake (*p* = 0.045, OR = 1.001, 95% CI: 1.000–1.002), white fish intake (*p* = 0.027, OR = 1.405, 95% CI: 1.039–1.899), and IVDD (Figure 8; Supplementary Table 6). The effect estimates from the other four models were consistent with this finding. These results suggest that excluding any single SNP does not significantly impact the overall findings, indicating the robustness of our MR study.

**Figure 9:**
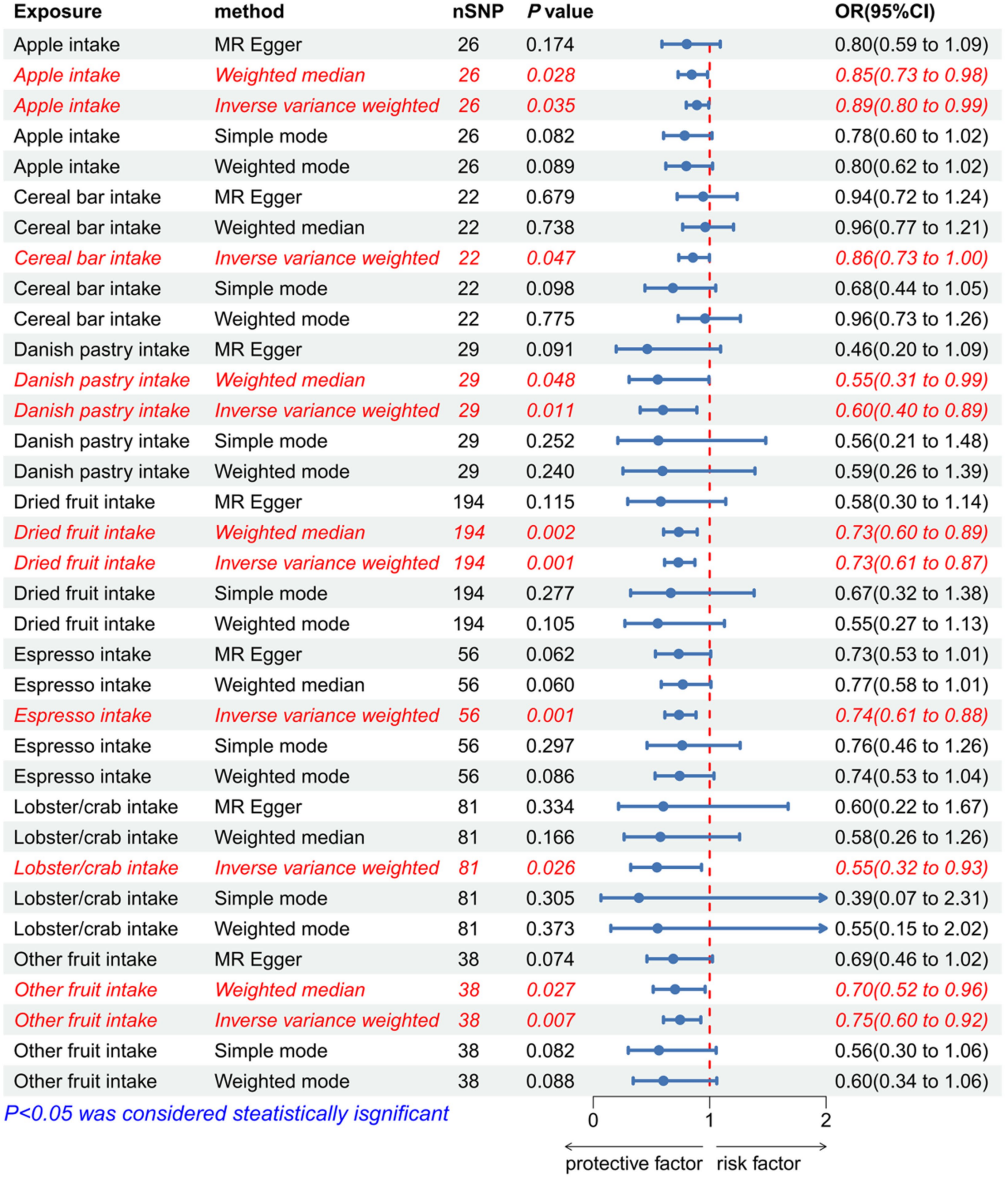
Forest plot to visualize the negative causal effect of dietary habits on IVDD. IVDD, intervertebral disc degeneration; OR, odds ratio; CI, confidence interval.

The IVW analysis revealed a negative correlation between apple intake (*p* = 0.035, OR = 0.890, 95% CI: 0.799–0.992), cereal bar intake (*p* = 0.047, OR = 0.856, 95% CI: 0.735–0.998), Danish pastry intake (*p* = 0.011, OR = 0.599, 95% CI: 0.403–0.890), dried fruit intake (*p* < 0.001, OR = 0.731, 95% CI: 0.613–0.873), espresso intake (*p* < 0.001, OR = 0.737, 95% CI: 0.615–0.883), lobster/crab intake (*p* = 0.026, OR = 0.547, 95% CI: 0.322–0.930), other fruit intake (*p* = 0.007, OR = 0.746, 95% CI: 0.603–0.924), and IVDD (Figure 9; Supplementary Table 6). The effect estimates from the four models were consistent with this finding. These results indicate that excluding any single SNP does not significantly impact the overall findings, indicating the robustness of our Mendelian randomization study. The *p*-value of IVW and MR-Egger regression analyses of alcohol intake frequency, alcohol intake compared with 10 years previously, standard tea intake, and dried fruit intake yielded Cochran’s Q statistics, which were less than 0.05, indicating a possible heterogeneity among the SNPs (Supplementary Table 3). Associations for Danish pastry, espresso, and lobster/crab did not survive Bonferroni correction (*p* > 0.0013) and are considered exploratory.

### A reverse MR analysis of the causal relationship between dietary patterns and IVDD

**Figure 10:**
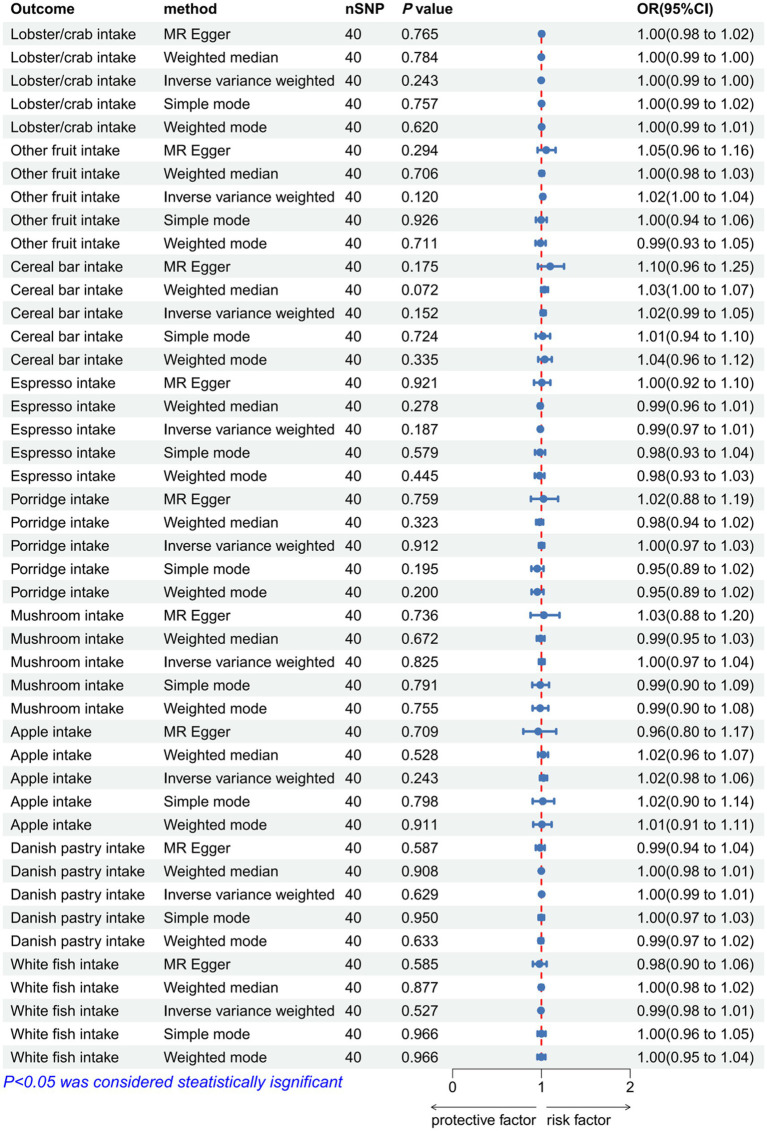
Forest plot to visualize the reverse Mendelian randomization causal effect of IVDD on dietary habits. IVDD, intervertebral disc degeneration; OR, odds ratio; CI, confidence interval.

After removing palindromic and ambiguous SNPs, SNPs without proxies, and SNPs in the wrong causal direction identified by the MR-Steiger filter, we extracted genome-wide significant SNPs as IVs. MR-PRESSO analysis did not detect any SNP outliers. The p-value of the MR-Egger regression was more than 0.05, indicating no pleiotropy among the included SNPs (Supplementary Table 1). The IVW analysis revealed no significant association in reverse MR analysis [lobster/crab intake (*p* = 0.243, OR = 0.998, 95% CI: 0.994–1.002), dried fruit intake (*p* = 0.463, OR = 1.006, 95% CI: 0.991–1.021), other fruit intake (*p* = 0.12, OR = 1.016, 95% CI: 0.996–1.036), cereal bar intake (*p* = 0.152, OR = 1.02, 95% CI: 0.993–1.048), espresso intake (*p* = 0.187, OR = 0.988, 95% CI: 0.97–1.006), porridge intake (*p* = 0.912, OR = 1.002, 95% CI: 0.972–1.032), standard tea intake (*p* = 0.242, OR = 0.018, 95% CI: 0–14.963), alcohol intake versus 10 years previously (*p* = 0.23, OR = 1.008, 95% CI: 0.995–1.021), mushroom intake (*p* = 0.825, OR = 1.004, 95% CI: 0.972–1.036), apple intake (*p* = 0.243, OR = 1.024, 95% CI: 0.984–1.064), Danish pastry intake (*p* = 0.629, OR = 1.003, 95% CI: 0.992–1.013), white fish intake (*p* = 0.527, OR = 0.995, 95% CI: 0.979–1.011), and alcohol intake frequency (*p* = 0.074, OR = 1.034, 95% CI: 0.997–1.073)] (Figure 10; Supplementary Table 7).

### Clinical validation of MR findings

The clinical cohort comprised 512 IVDD patients (mean age 56.3 ± 10.2 years, 54.7% female) and 512 controls (mean age 55.8 ± 9.8 years, 53.9% female). Compared to controls, IVDD patients had significantly higher WC (98.2 ± 12.5 cm vs. 91.4 ± 11.8 cm, *p* < 0.001) and a higher prevalence of frequent mushroom consumption (≥3 times/week: 38.5% vs. 28.9%, *p* = 0.003). In the multivariate logistic regression analysis, larger WC (OR = 1.32 per 5 cm increase, 95% CI: 1.10–1.58, *p* = 0.003) and frequent mushroom intake (OR = 1.28, 95% CI: 1.05–1.56, *p* = 0.015) were independently associated with higher IVDD risk. Conversely, higher fruit intake (≥2 servings/day) was protective (OR = 0.76, 95% CI: 0.62–0.93, *p* = 0.009). No significant associations were found for BMI, HC, or WHR-BMI after adjustment, consistent with MR results (Table 2).

## Discussion

The relationship between obesity measurement indicators and IVDD remains inconclusive, although it has been examined in recent studies. Several clinical studies have explored the relationship between obesity related indicators such as BMI, WC, ASAT, and IVDD, but these research results may have certain limitations due to the insufficient samples. We use MR analysis to investigate the association between BMI, WC, HC, ASAT, WHR, WHR-BMI, and IVDD, and our results suggest that genetically predicted WC and ASAT are two important risk factors associated with IVDD, while BMI, HC, and WHR-BMI are revealed to have no causal relationship with IVDD, and the relationship between WHR and IVDD could not be elucidated. Consequently, the relationship between dietary patterns and IVDD remains inconclusive since there are only a few research studies on it. The findings of the clinical analysis could have some limitations owing to the use of the observational study method. We used MR analysis to investigate the association between dietary patterns and IVDD. Our results indicate that following nine dietary patterns, such as mushroom intake, porridge intake, white fish intake, apple intake, cereal bar intake, Danish pastry intake, espresso intake, lobster/crab intake, and other fruit intake, are closely related to the occurrence of IVDD. However, the results of alcohol intake frequency, alcohol intake compared with 10 years previously, standard tea intake, and dried fruit intake show heterogeneity. At present, we are the first ones to demonstrate the relationship between dietary patterns and IVDD using the MR method.

IVDD is a highly prevalent clinical condition that can lead to lumbar pain and progressive physical disability. Currently, there is no definitive treatment for IVDD, prompting several scholars to explore its pathogenesis (36). Numerous clinical studies have investigated the risk factors of IVDD in hopes of preventing or slowing its progression. It is generally accepted that the progression of IVDD is primarily due to structural damage to the IVD (37). The IVD is a crucial component supporting the body’s structure, and excessive mechanical forces often lead to its damage. Recent research has found that metabolic disturbances seem to have a more significant impact on IVDD than biomechanical changes (13). Metabolic disturbances can cause various diseases, such as diabetes, obesity, hyperlipidemia, and more. The majority of researchers have used MR methods to explore the risk factors of IVDD. Beyond risk identification, emerging therapeutic strategies targeting the metabolic-inflammatory axis show promise. Engineered exosomes loaded with salvianolic acid A, incorporated into CaCO₃/chitosan hydrogel, can specifically target cartilage endplate cells, simultaneously mitigating iron overload-induced ROS and neutralizing acidic microenvironment—addressing both cellular metabolism and nutritional factors identified in our MR study (38). Microbial taxa and their metabolic pathways have a causal relationship with IVDD (39, 40); high BMI has a causal relationship with the risk of IVDD; BMI and type 2 diabetes mellitus-mediated television watching contribute to the progression of IVDD (41). These studies have consistently found that obesity has a significant impact on IVDD.

Exploratory dietary associations require cautious interpretation, while WC, mushroom, and fruit intake associations survived Bonferroni correction and align with clinical validation. Several dietary patterns (e.g., Danish pastry, espresso and lobster/crab) exhibited nominal protective effects that appear counterintuitive. These associations (all *p* > 0.0013) should be considered exploratory for three reasons: (1) they lack direct mechanistic support in IVD biology; (2) genetic instruments may capture broad lifestyle confounding (e.g., espresso consumption proxies for higher socioeconomic status); and (3) they did not reach multiple-testing-corrected significance thresholds. We emphasize that these observations must not inform dietary recommendations until validated by functional studies (e.g., ex vivo disc organ culture models) and prospective cohorts.

It is widely recognized in the scientific community that obesity is a risk factor for IVDD; however, there is still controversy on which detection indicator is highly correlated with the occurrence of IVDD. BMI, as an indicator of whether a body is obese, is associated with multiple diseases, including depression (42), abdominal hernia (43), cardiac disease (44), and so on. Clinical research has reported that, in patients, especially women who suffer from LBP, higher BMI could lead to more severe IVDD (45). Prior MR studies have reported associations between higher BMI and increased low back pain risk (3, 39). However, our FinnGen analysis provides robust genetic evidence that central obesity metrics—specifically WC and ASAT, not BMI—are the key causal factors for IVDD. This finding aligns with clinical evidence indicating that subcutaneous fat tissue thickness and ASAT outperform BMI as predictors in IVDD patients (44, 45). Moreover, the association between WC and IVDD extends to early adulthood (46), underscoring abdominal adiposity as a critical risk factor across the lifespan.

In our research, we first examined the relationship between BMI and IVDD via MR analysis. The results show that BMI had no causal relationship with IVDD because the *p-*values in different analyses were greater than 0.05. This finding indicates that BMI is not a key factor affecting the process of IVDD. Then, we detected whether WC, HC, and ASAT could be the key factors affecting IVDD. Consistent with previous clinical results, our study found a positive correlation between WC, ASAT, and IVDD, but there was no significant correlation between HC and IVDD. This finding indicated that, in patients with larger WC, the greater the volume of ASAT, the more likely IVDD is to occur. The adipose tissue in the waist and hip plays an important role in the occurrence and development of IVDD. Some studies have shown results similar to ours, which indicates that the abdominal fat mass parameters, such as WC, total fat mass, and visceral fat mass, are significantly related to LBP rather than BMI (37). Abdominal fat was reported to place a mechanical load on the spine (10), and this could explain why increased WC and ASAT lead to the occurrence of IVDD. Some researchers suggest that WHR may be a more appropriate index for some diseases than BMI, as BMI does not efficiently reflect the body fat distribution of one person (46). Then, we detected the relationship between WHR, WHR-BMI, and IVDD. WHR shows a positive correlation with IVDD, but the heterogeneity of MR was not an ideal result. We could not ensure the relationship between them. However, there was no significant correlation between WHR–BMI and IVDD. The relationship between hip traits and IVDD needs further exploration and investigation.

The majority of clinical studies have already reported that various lifestyle factors can influence the development of IVDD (47). It is well known that obesity is a risk factor for IVDD; therefore, controlling weight is an effective method to alleviate IVDD (3). Adopting a reasonable diet is the best choice for weight management, and it is highly necessary to explore the impact of different dietary patterns on IVDD.

With the deepening of research on various diets, researchers are increasingly realizing that diet can have important effects on the human body; for example, increased oily fish consumption leads to higher DHA in the human body (48); increased vegetable consumption raises omega-6 in human body (49); and increased coffee consumption elevates total and LDL cholesterol in human body (50). Several studies have found that different dietary patterns are closely related to the occurrence of cancer. Researchers suggest that dried fruits and oily fish consumption could decrease the occurrence of breast cancer (51); intake of carbohydrate and sugar reduced risk of endometrial cancer (52); cheese, dried fruit, and beer intake are significantly associated with the risk of lung cancer (53).

Limiting calorie intake is a common method for controlling weight, and selecting an appropriate dietary pattern requires further exploration (54). We have found that increased mushroom intake, porridge intake, and white fish intake had a positive correlation with IVDD, and increased apple intake, cereal bar intake, Danish pastry intake, espresso intake, lobster/crab intake, and other fruit intake had a negative correlation with IVDD.

Some researchers have stated that higher mushroom intake is associated with a lower risk of cancer and lower odds of depression (55, 56). However, our results have found that higher mushroom intake could increase the risk of IVDD. Therefore, mushroom intake is a complex condition for overall health. Similarly, the observed “protective” effects of espresso, Danish pastry, and lobster/crab appear counterintuitive but may be explained by two hypotheses: (1) these foods may act as proxy for healthier lifestyles (e.g., coffee consumption correlates with higher physical activity) and (2) specific bioactive components (e.g., chlorogenic acid in coffee and omega-3 fatty acids in seafood) may exert anti-inflammatory effects on the intervertebral disc. However, these observations remain exploratory, lack mechanistic validation, and should be interpreted as hypothesis-generating rather than definitive dietary recommendations. Porridge was reported to accelerate intestinal motility (57), and we believe that this could lead to an increased accumulation of body fat and then increase the risk of IVDD. High-temperature cooking methods for white fish may increase prostate cancer risk (58); our research had the same results.

Fruits, especially apples, are rich in nutrients, and studies had provided much support that fruits could prevent various chronic diseases (59). Espresso is made into an extremely popular beverage that we often drink. Some studies have reported that it could reduce the risk of cardiovascular and total mortality (60). Our results are consistent with them.

To address the need for empirical validation of MR-derived associations, we conducted an independent clinical retrospective study. The clinical findings consistently supported the MR results: larger WC and frequent mushroom intake were independently associated with a higher risk of IVDD, while higher fruit intake exhibited a protective effect. This convergence of evidence from genetic instrumental variable analysis and observational clinical data significantly strengthens the causal inference and underscores the biological and clinical relevance of abdominal adiposity and specific dietary habits in the etiology of IVDD. The lack of association between BMI and HC in both analyses further suggests that central obesity, rather than overall weight or hip adiposity, is a critical risk factor.

However, our study has several limitations. First, our analysis was performed using the IEU OpenGWAS database, which lacks age and gender information. Second, the clinical validation was restricted to WC, mushroom, and fruit intake based on pragmatic methodological considerations: (1) Statistical power: post-hoc calculations revealed that exposures with <5% consumption frequency in our Chinese cohort (lobster/crab 4.2%, Danish pastry 7.8%) would require >2,000 participants to achieve 80% power (*α* = 0.05, OR = 0.7); (2) Cultural relevance: items such as cereal bars and espresso have low consumption frequency (<8%) and poor recall accuracy, limiting FFQ reliability; (3) Effect size prioritization: we focused on associations surviving Bonferroni correction with OR>1.2 or <0.8. Future multi-center studies with culturally adapted dietary assessments are needed to validate the full spectrum of associations.

Other MR-significant foods (e.g., Danish pastry and espresso) had low consumption frequency in our Chinese cohort (<5%), precluding reliable statistical validation. Future multi-center studies with larger sample sizes and culturally adapted dietary assessments are needed to validate the full spectrum of dietary associations. Third, as a retrospective case–control study, dietary data collection via FFQ is subject to recall bias. Third, the retrospective case–control design introduces two critical limitations: (1) Differential recall bias—IVDD patients may systematically over-report suspected risk foods. While we used a validated FFQ with face-to-face interviews and visual portion aids, bias cannot be fully eliminated without a prospective design and (2) the lack of Fireman grading for IVDD severity represents a significant limitation—we could not assess dose–response relationships or differentiate early versus advanced degeneration. This binary disease classification may obscure non-linear associations and warrants future cohorts integrating quantitative MRI grading with food diaries. Fourth, our experiment did not investigate the effect of fat and muscle on IVDD. Finally, our experiment did not have the evidence of clinical samples. In subsequent studies, we can add some clinical samples to validate the results above and do some experiments to explore their internal connections. Particularly, our MR framework establishes the causal role of abdominal obesity (mechanical + metabolic), but the molecular crosstalk between mechanical stress and metabolic signaling remains unclear. A recent study shows that cyclic mechanical tension promotes nucleus pulposus cell-derived exosomes carrying miR-8485, activating the Wnt/*β*-catenin pathway via GSK-3β inhibition, thereby delaying IVDD (61). This finding bridges our epidemiological observation with cellular pathogenesis, suggesting that abdominal obesity may exacerbate IVDD not only through systemic inflammation but also by altering mechanosensitive exosomal signaling. Future studies should integrate MR-derived causal exposures with mechanistic investigations of exosome-mediated mechanometabolic coupling.

Additionally, generalizability is limited to European-ancestry populations, as our GWAS data lacked diversity. The median age of the FinnGen cohort was 58 years; therefore, the results may not be applicable to younger adults where mechanical factors dominate. Furthermore, our results primarily reflect lumbar IVDD (90% of FinnGen cases), and applicability to cervical degeneration remains uncertain. Future MR studies in non-European ancestries, age-stratified analyses, and anatomic site-specific investigations are warranted to broaden the inferential boundaries.

## Conclusion

Our bidirectional MR study provides genetic evidence supporting a causal role of abdominal obesity—specifically indicated by waist circumference (WC) and abdominal subcutaneous adipose tissue (ASAT)—in the development of intervertebral disc degeneration (IVDD), independent of general adiposity measured by body mass index (BMI). In parallel, we identified several dietary patterns (increased intake of mushrooms, porridge, and white fish as risk factors, and higher consumption of apple, cereal bar, Danish pastry, espresso, lobster/crab, and other fruits as protective factors) that are causally linked to IVDD risk.

Critically, these MR-derived associations were substantiated through an independent clinical retrospective validation. Our clinical cohort confirmed that larger WC and frequent mushroom intake are associated with a higher risk of IVDD, while greater fruit consumption is protective. This convergence of evidence from genetic instrumental variable analysis and real-world clinical data significantly strengthens the causal inference and enhances the translational relevance of our findings.

In clinical practice, waist-centric adiposity measures (WC and ASAT), rather than BMI or hip circumference, should be prioritized for risk assessment in patients with low back pain. Furthermore, our identification of specific dietary patterns as modifiable risk factors should be integrated with other lifestyle interventions. Notably, a systematic review of 22 randomized controlled trials demonstrated that traditional Chinese exercises such as Baduanjin, Yijinjing, Tai Chi, and Wuqinxi significantly improve pain and disability in elderly patients with lumbar disc herniation (62). This evidence, combined with our MR-derived dietary recommendations, supports a comprehensive lifestyle modification strategy encompassing both waist-targeted weight management and specific dietary patterns for IVDD prevention and management. While MR analysis robustly suggests causality and our clinical validation supports these associations, further prospective and mechanistic studies are warranted to elucidate the underlying biological pathways and to inform precise public health interventions.

## Data Availability

The original contributions presented in the study are included in the article/Supplementary material, further inquiries can be directed to the corresponding authors.

## References

[1] Fenn J Olby NJ Canine Spinal Cord Injury C. Classification of intervertebral disc disease. Front Vet Sci. (2020) 7:579025. doi: 10.3389/fvets.2020.579025 33134360 PMC7572860

[2] Samanta A Lufkin T Kraus P. Intervertebral disc degeneration-current therapeutic options and challenges. Front Public Health. (2023) 11:1156749. doi: 10.3389/fpubh.2023.1156749, 37483952 PMC10359191

[3] Zhou J Mi J Peng Y Han H Liu Z. Causal associations of obesity with the intervertebral degeneration, low back pain, and sciatica: a two-sample mendelian randomization study. Front Endocrinol (Lausanne). (2021) 12:740200. doi: 10.3389/fendo.2021.740200, 34956075 PMC8692291

[4] Li Z Chen X Xu D Li S Chan MTV Wu WKK. Circular RNAs in nucleus pulposus cell function and intervertebral disc degeneration. Cell Prolif. (2019) 52:e12704. doi: 10.1111/cpr.12704, 31621141 PMC6869348

[5] Xiang H Zhao W Jiang K He J Chen L Cui W . Progress in regulating inflammatory biomaterials for intervertebral disc regeneration. Bioact Mater. (2024) 33:506–31. doi: 10.1016/j.bioactmat.2023.11.021, 38162512 PMC10755503

[6] Francisco V Pino J Gonzalez-Gay MA. A new immunometabolic perspective of intervertebral disc degeneration. Nat Rev Rheumatol. (2022) 18:47–60. doi: 10.1038/s41584-021-00719-9 34845360

[7] Liu W Ma Z Wang Y Yang J. Multiple nano-drug delivery systems for intervertebral disc degeneration: current status and future perspectives. Bioact Mater. (2023) 23:274–99. doi: 10.1016/j.bioactmat.2022.11.006, 36439088 PMC9679278

[8] Tessier S Tran VA Ottone OK Novais EJ Doolittle A DiMuzio M . TonEBP-deficiency accelerates intervertebral disc degeneration underscored by matrix remodeling, cytoskeletal rearrangements, and changes in proinflammatory gene expression. Matrix Biol. (2020) 87:94–111. doi: 10.1016/j.matbio.2019.10.007, 31707045 PMC7078052

[9] Vergroesen PP Kingma I Emanuel KS Hoogendoorn RJ Welting TJ van Royen B . Mechanics and biology in intervertebral disc degeneration: a vicious circle. Osteoarthr Cartil. (2015) 23:1057–70. doi: 10.1016/j.joca.2015.03.028, 25827971

[10] Dario AB Ferreira ML Refshauge KM Lima TS Ordoñana JR Ferreira PH. The relationship between obesity, low back pain, and lumbar disc degeneration when genetics and the environment are considered: a systematic review of twin studies. Spine J. (2015) 15:1106–17. doi: 10.1016/j.spinee.2015.02.001, 25661432

[11] Nijs J D'Hondt E Clarys P Deliens T Polli A Malfliet A . Lifestyle and chronic pain across the lifespan: an inconvenient truth? PM R. (2020) 12:410–9. doi: 10.1002/pmrj.12244, 31437355

[12] Zhang W Li Y Shao P Du Y Zhao K Zhan J . Association of weight-adjusted waist index and body mass index with chronic low back pain in American adults: a retrospective cohort study and predictive model development based on machine learning algorithms (NHANES 2009-2010). Front Public Health. (2025) 13:1617732. doi: 10.3389/fpubh.2025.1617732, 40717955 PMC12289706

[13] Guo W Li BL Zhao JY Li XM Wang LF. Causal associations between modifiable risk factors and intervertebral disc degeneration. Spine J. (2024) 24:195–209. doi: 10.1016/j.spinee.2023.10.021, 37939919

[14] Taba N Valge HK Metspalu A Esko T Wilson JF Fischer K . Mendelian randomization identifies the potential causal impact of dietary patterns on circulating blood metabolites. Front Genet. (2021) 12:738265. doi: 10.3389/fgene.2021.738265, 34790224 PMC8592281

[15] Wang C Zhu Y Liu Z. Causal associations of obesity related anthropometric indicators and body compositions with knee and hip arthritis: a large-scale genetic correlation study. Front Endocrinol (Lausanne). (2022) 13:1011896. doi: 10.3389/fendo.2022.1011896 36246900 PMC9556900

[16] Berikol G Eksi MS Aydin L. Subcutaneous fat index: a reliable tool for lumbar spine studies. Eur Radiol. (2022) 32:6504–13. doi: 10.1007/s00330-022-08775-7, 35380225

[17] Kilinc RM Can FI. The effect of intraabdominal visceral and subcutaneous adipose volume and muscle volume on lumbar vertebrae degeneration. Cureus. (2023) 15:e35940. doi: 10.7759/cureus.35940 36911579 PMC9999032

[18] Chong M Sjaarda J Pigeyre M Mohammadi-Shemirani P Lali R Shoamanesh A . Novel drug targets for ischemic stroke identified through Mendelian randomization analysis of the blood proteome. Circulation. (2019) 140:819–30. doi: 10.1161/CIRCULATIONAHA.119.040180, 31208196

[19] Okada E Matsumoto M Ichihara D. Aging of the cervical spine in healthy volunteers: a 10-year longitudinal magnetic resonance imaging study. Spine (Phila Pa 1976). (2009) 34:706–12. doi: 10.1097/BRS.0b013e3181a16123 19333104

[20] Castano-Betancourt MC Oei L Rivadeneira F de Schepper EIT Hofman A Bierma-Zeinstra S . Association of lumbar disc degeneration with osteoporotic fractures; the Rotterdam study and meta-analysis from systematic review. Bone. (2013) 57:284–9. doi: 10.1016/j.bone.2013.08.004, 23958823

[21] Liu M Jiang Y Wedow R Li Y. Association studies of up to 1.2 million individuals yield new insights into the genetic etiology of tobacco and alcohol use. Nat Genet. (2019) 51:237–44. doi: 10.1038/s41588-018-0307-5, 30643251 PMC6358542

[22] Lee JJ Wedow R Okbay A Kong E. Gene discovery and polygenic prediction from a genome-wide association study of educational attainment in 1.1 million individuals. Nat Genet. (2018) 50:1112–21. doi: 10.1038/s41588-018-0147-3, 30038396 PMC6393768

[23] Hemani G Zheng J Elsworth B. The MR-base platform supports systematic causal inference across the human phenome. eLife. (2018) 7:e34408. doi: 10.7554/eLife.34408 29846171 PMC5976434

[24] Kurki MI Karjalainen J Palta P Sipilä TP. FinnGen provides genetic insights from a well-phenotyped isolated population. Nature. (2023) 613:508–18. doi: 10.1038/s41586-022-05473-8, 36653562 PMC9849126

[25] Richardson TG Sanderson E Palmer TM Ala-Korpela M Ference BA Davey Smith G . Evaluating the relationship between circulating lipoprotein lipids and apolipoproteins with risk of coronary heart disease: a multivariable Mendelian randomisation analysis. PLoS Med. (2020) 17:e1003062. doi: 10.1371/journal.pmed.1003062, 32203549 PMC7089422

[26] Shungin D Winkler TW Croteau-Chonka DC Ferreira T Locke AE. New genetic loci link adipose and insulin biology to body fat distribution. Nature. (2015) 518:187–96. doi: 10.1038/nature14132, 25673412 PMC4338562

[27] Tachmazidou I Hatzikotoulas K Southam L. Identification of new therapeutic targets for osteoarthritis through genome-wide analyses of UK biobank data. Nat Genet. (2019) 51:230–6. doi: 10.1038/s41588-018-0327-1 30664745 PMC6400267

[28] Jiang X O'Reilly PF Aschard H. Genome-wide association study in 79,366 European-ancestry individuals informs the genetic architecture of 25-hydroxyvitamin D levels. Nat Commun. (2018) 9:260. doi: 10.1038/s41467-018-06623-8 29343764 PMC5772647

[29] Burgess S Butterworth A Thompson SG. Mendelian randomization analysis with multiple genetic variants using summarized data. Genet Epidemiol. (2013) 37:658–65. doi: 10.1002/gepi.21758 24114802 PMC4377079

[30] Lyon MS Andrews SJ Elsworth B. The variant call format provides efficient and robust storage of GWAS summary statistics. Genome Biol. (2021) 22:32. doi: 10.1186/s13059-021-02248-2 33441155 PMC7805039

[31] Burgess S Thompson SG. Interpreting findings from Mendelian randomization using the MR-egger method. Eur J Epidemiol. (2017) 32:377–89. doi: 10.1007/s10654-017-0255-x, 28527048 PMC5506233

[32] Bowden J Davey Smith G Burgess S. Mendelian randomization with invalid instruments: effect estimation and bias detection through egger regression. Int J Epidemiol. (2015) 44:512–25. doi: 10.1093/ije/dyv080, 26050253 PMC4469799

[33] Bowden J Davey Smith G Haycock PC Burgess S. Consistent estimation in Mendelian randomization with some invalid instruments using a weighted median estimator. Genet Epidemiol. (2016) 40:304–14. doi: 10.1002/gepi.21965, 27061298 PMC4849733

[34] Tan JS Ren JM Fan L Wei Y Hu S Zhu SS . Genetic predisposition of anti-cytomegalovirus immunoglobulin G levels and the risk of 9 cardiovascular diseases. Front Cell Infect Microbiol. (2022) 12:884298. doi: 10.3389/fcimb.2022.884298, 35832381 PMC9272786

[35] Verbanck M Chen CY Neale B Do R. Detection of widespread horizontal pleiotropy in causal relationships inferred from Mendelian randomization between complex traits and diseases. Nat Genet. (2018) 50:693–8. doi: 10.1038/s41588-018-0099-7, 29686387 PMC6083837

[36] Zhou D Song C Mei Y. A review of Duhuo Jisheng decoction mechanisms in intervertebral disc degeneration in vitro and animal studies. J Orthop Surg Res. (2023) 18:436. doi: 10.1186/s13018-023-04027-8 37322524 PMC10273736

[37] Baek S Park HW Kim G. Associations between trunk muscle/fat composition, narrowing lumbar disc space, and low Back pain in middle-aged farmers: a cross-sectional study. Ann Rehabil Med. (2022) 46:122–32. doi: 10.5535/arm.21201, 35793901 PMC9263327

[38] Zhan J Cui Y Zhang P. Cartilage endplate-targeted engineered exosome releasing and acid neutralizing hydrogel reverses intervertebral disc degeneration. Adv Healthc Mater. (2025) 14:e2403315. doi: 10.1002/adhm.202403315 39555665

[39] Fang M Liu W Wang Z Li J Hu S Li Z . Causal associations between gut microbiota with intervertebral disk degeneration, low back pain, and sciatica: a Mendelian randomization study. Eur Spine J. (2024) 33:1424–39. doi: 10.1007/s00586-024-08131-x, 38285276

[40] Geng Z Wang J Chen G. Gut microbiota and intervertebral disc degeneration: a bidirectional two-sample Mendelian randomization study. J Orthop Surg Res. (2023) 18:601. doi: 10.1186/s13018-023-04305-5 37580794 PMC10424333

[41] Qiu Y Wei X Tao Y Song B Wang M Yin Z . Causal association of leisure sedentary behavior and cervical spondylosis, sciatica, intervertebral disk disorders, and low back pain: a Mendelian randomization study. Front Public Health. (2024) 12:1284594. doi: 10.3389/fpubh.2024.1284594, 38322127 PMC10844448

[42] Lo DF Thompson J. Body mass index on perinatal depression: a critical viewpoint. Eur Psychiatry. (2023) 66:e72. doi: 10.1192/j.eurpsy.2023.136 37772364 PMC10594364

[43] Li Z Xia L Li X Guan Y He H Jin L. Body mass index and the risk of abdominal hernia: a Mendelian randomization study. Hernia. (2023) 27:423–9. doi: 10.1007/s10029-022-02703-w, 36441335

[44] Lee H Shin H Oh J Lim TH Kang BS Kang H . Association between body mass index and outcomes in patients with return of spontaneous circulation after out-of-hospital cardiac arrest: a systematic review and meta-analysis. Int J Environ Res Public Health. (2021) 18:8389. doi: 10.3390/ijerph18168389, 34444142 PMC8394455

[45] Ozcan-Eksi EE Turgut VU Kucuksuleymanoglu D Eksi MS. Obesity could be associated with poor paraspinal muscle quality at upper lumbar levels and degenerated spine at lower lumbar levels: is this a domino effect? J Clin Neurosci. (2021) 94:120–7. doi: 10.1016/j.jocn.2021.10.005, 34863425

[46] Haufs MG Zöllner YF. Waist-hip ratio more appropriate than body mass index. Dtsch Arztebl Int. (2020) 117:659. doi: 10.3238/arztebl.2020.1007 PMC7829451 33357347

[47] Agius R Galea R Fava S. Bone mineral density and intervertebral disc height in type 2 diabetes. J Diabetes Complicat. (2016) 30:644–50. doi: 10.1016/j.jdiacomp.2016.01.021, 26954485

[48] Horrocks LA Yeo YK. Health benefits of docosahexaenoic acid (DHA). Pharmacol Res. (1999) 40:211–25. 10479465 10.1006/phrs.1999.0495

[49] Kornsteiner M Singer I Elmadfa I. Very low n-3 long-chain polyunsaturated fatty acid status in Austrian vegetarians and vegans. Ann Nutr Metab. (2008) 52:37–47. doi: 10.1159/000118629, 18305382

[50] Poole R Kennedy OJ Roderick P. Coffee consumption and health: umbrella review of meta-analyses of multiple health outcomes. BMJ. (2017) 359:j5024. doi: 10.1136/bmj.j5024 29167102 PMC5696634

[51] Wang X Chen L Cao R. Associations of 10 dietary habits with breast cancer: a Mendelian randomization study. Front Nutr. (2023) 10:1215220. doi: 10.3389/fnut.2023.1215220 38075235 PMC10702979

[52] Wang X Glubb DM O'Mara TA. Dietary factors and endometrial cancer risk: a Mendelian randomization study. Nutrients. (2023) 15:2627. 36771310 10.3390/nu15030603 PMC9920466

[53] Yan H Jin X Zhang C Zhu C He Y Du X . Associations between diet and incidence risk of lung cancer: a Mendelian randomization study. Front Nutr. (2023) 10:1149317. doi: 10.3389/fnut.2023.1149317, 37063327 PMC10102585

[54] Jiang K Zhang Z Fullington LA. Dietary patterns and obesity in Chinese adults: a systematic review and meta-analysis. Nutrients. (2022) 14:4833. doi: 10.3390/nu14224833 36432596 PMC9698822

[55] Ba DM Gao X Al-Shaar L Muscat JE Chinchilli VM Beelman RB . Mushroom intake and depression: a population-based study using data from the US National Health and nutrition examination survey (NHANES), 2005-2016. J Affect Disord. (2021) 294:686–92. doi: 10.1016/j.jad.2021.07.080 34333177

[56] Ba DM Ssentongo P Beelman RB Muscat J Gao X Richie JP. Higher mushroom consumption is associated with lower risk of Cancer: a systematic review and Meta-analysis of observational studies. Adv Nutr. (2021) 12:1691–704. doi: 10.1093/advances/nmab015, 33724299 PMC8483951

[57] Chen Y Zhang R Xu J Ren Q. Alteration of intestinal microflora by the intake of millet porridge improves gastrointestinal motility. Front Nutr. (2022) 9:965687. doi: 10.3389/fnut.2022.965687, 36071942 PMC9442030

[58] Joshi AD John EM Koo J Ingles SA Stern MC. Fish intake, cooking practices, and risk of prostate cancer: results from a multi-ethnic case-control study. Cancer Causes Control. (2012) 23:405–20. doi: 10.1007/s10552-011-9889-2, 22207320

[59] Slavin JL Lloyd B. Health benefits of fruits and vegetables. Adv Nutr. (2012) 3:506–16. doi: 10.3945/an.112.002154, 22797986 PMC3649719

[60] Ruggiero E Di Castelnuovo A Costanzo S. Daily coffee drinking is associated with lower risks of cardiovascular and total mortality in a general Italian population: results from the Moli-sani study. J Nutr. (2021) 151:395–404. doi: 10.1093/jn/nxaa363 33382422

[61] Zhang W Zhang P Zhan J. Role of exosomes from nucleus Pulposus cells in attenuating intervertebral disc degeneration by inhibiting nucleus Pulposus cell apoptosis via the miR-8485/GSK-3beta/Wnt/beta-catenin signaling axis. Curr Mol Med. (2025) doi: 10.2174/1566524025666250331141325 PMC13555773 40600527

[62] Zhang W Wang G Xie R Zhan J Zhu L Wan C . Traditional Chinese exercises on pain and disability in middle-aged and elderly patients with lumbar disc herniation: a systematic review and meta-analysis of randomized controlled trials. Front Med. (2023) 10:1265040. doi: 10.3389/fmed.2023.1265040, 38020108 PMC10663407

